# Differences in circulating levels of non-classical monocytes and associated caspase 1 activation during *Staphylococcus aureus* and *Escherichia coli* bacteremia

**DOI:** 10.1080/21505594.2026.2730393

**Published:** 2026-11-01

**Authors:** Johan Courjon, Renaud Scussel, Eva Mellan, Romain Lotte, Raymond Ruimy, Elisa Demonchy, Karine Risso, Irit Touitou, Pierre-Marie Bertrand, Philippe de Swart, Alexandre Robert, Julie Contenti, Jean Dellamonica, Mathieu Jozwiak, Géraldine Gonfrier, Patrick Munro, Anne Doye, Céline Loubatier, Orane Visvikis, Paul Chaintreuil, Arnaud Jacquel, Océane Dufies, Michel Carles, Laurent Boyer

**Affiliations:** a Inserm, C3M, Université Côte d’Azur, INSERM, C3M, Nice, France; b CHU Nice, Université Côte d’Azur, CHU Nice, Nice, France; c Réanimation, Centre Hospitalier de Cannes, Cannes, France; d Réanimation, Centre Hospitalier d’Antibes, Antibes, France; e Pediatrics, Harvard Medical School, Boston, MA, USA

**Keywords:** Bacteremia, S. aureus, E. coli, caspase 1, non-classical monocytes, intermediate monocytes

## Abstract

Bloodstream infections (BSI) caused by *Escherichia coli* and *Staphylococcus aureus* are frequent and are associated with substantial mortality rate. The host immune response major determinant of the clinical outcomes during BSI. Here, we investigated the innate immune response, by characterizing peripheral blood myeloid cell subsets and caspase-1 activation as a surrogate marker of inflammasome activation in patients with *E. coli* or *S. aureus* bacteremia. We prospectively enrolled patients with monomicrobial bacteremia and analyzed peripheral blood samples by flow cytometry. We observed distinct alterations in myeloid cell composition and caspase-1 activation associated with *E. coli* bacteremia, including an expansion in intermediate and non-classical monocytes, and increased caspase-1 activation in non-classical monocytes. In contrast, these changes were not observed in patients with *S. aureus* bacteremia. Additionally, we found differences in myeloid cell subset frequencies and caspase-1 activation between patients with and without sepsis, revealing distinct profiles associated with disease severity. Importantly, the differences in myeloid cell subsets and caspase-1 activation between *E. coli* and *S. aureus* bacteremia were primarily attributable to the infecting bacteria rather than disease severity. Furthermore, our findings suggest that eosinophil counts, and caspase-1 activation may serve as potential markers to distinguish between bacterial BSI and viral infections. Overall, our study highlights the importance of considering both the infecting pathogen and disease severity when studying the host immune response during bacteremia and suggests that potential innate immune biomarkers may improve the diagnosis and clinical management of BSI.

## BRIEF COMMENTARY

**Background:**

In translational immunology studies conducted in intensive care units on sepsis, microbiological data are often inconsistently reported, and the investigation of innate or adaptive immune responses based on the etiologic agent involved is scarce.

**Translational significance:**

Here, we show that during bacteremia, the abundance of specific subtypes of circulating myeloid cells, such as non-classical monocytes, as well as their caspase 1 activation, differ depending on the causative bacteria, highlighting pathogen-specific immune signatures with potential diagnostic and therapeutic relevance.

## GRAPHICAL ABSTRACT

**Figure:**
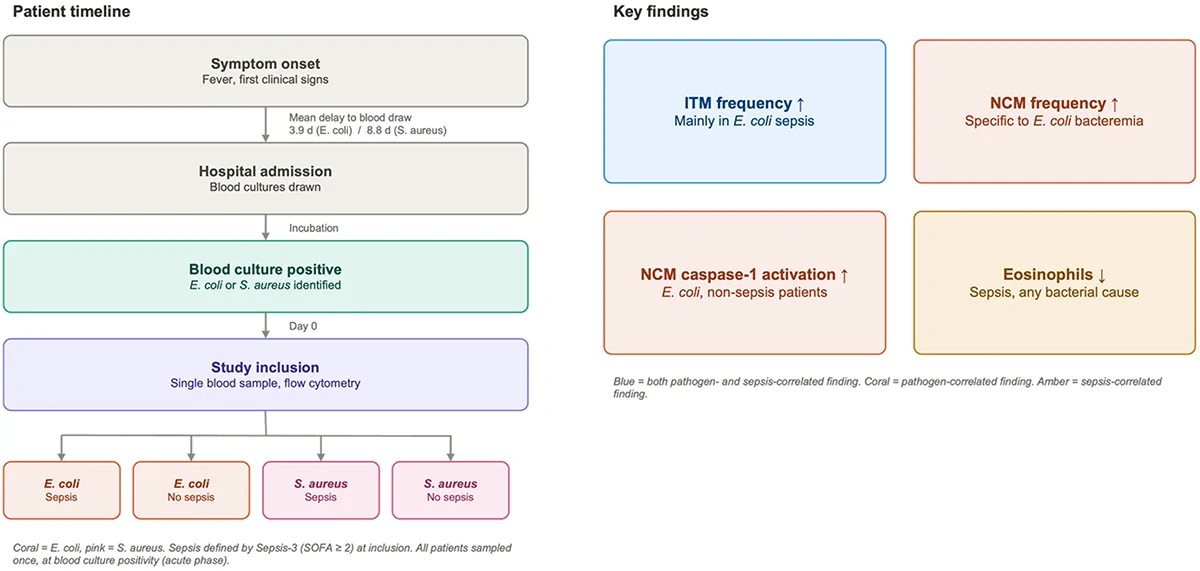

## Introduction

Bloodstream infections (BSI) are defined by the vascular dissemination of one or more bacteria from an initial source of infection. As an example, in a region such as Ontario in Canada, the incidence of BSI was estimated at 150 per 100,000 population per year in 2017, based on data from both hospital and community settings [1]. *Escherichia coli* and *Staphylococcus aureus* were the most frequently identified pathogens, consistent with findings typically reported in other studies [2]. In a nationwide Israeli study including 25,376 cases of BSI (both community- and hospital-onset cases) the all-cause mortality rate was 25.6% at 30 days and reached 46.4% at one year [3]. BSI range from uncomplicated *E. coli* bacteremia associated with urinary tract infection (UTI) to persistent *S. aureus* bacteremia (SAB) despite appropriate antibiotics and source control [4]. Such heterogeneous clinical situations emphasize the crucial role of host response during BSI.

The intensity of the immune response during BSI is determined, in part, by the innate immune sensing of pathogens. The innate immune recognition system of bacteria relies on pattern recognition receptors (PRRs). PRRs are conserved proteins that recognize pathogen-associated molecular patterns (PAMPs) derived from microorganisms [5]. Bacterial PAMPs such as lipopolysaccharides, lipoteichoic acid, or muramyl dipeptide have been shown to activate PRRs and their downstream signaling pathways [6]. Among the PRRs, nucleotide-binding domain leucine-rich repeat containing proteins (NLRs) are cytosolic receptors that control the maturation of interleukin-1β (IL-1β) and IL-18. NLRs bind caspase-1 either directly or indirectly via the adaptor protein ASC to form supramolecular complexes called inflammasomes. Upon infection, inflammasomes assemble, promoting caspase-1 activation and the subsequent cleavage of pro-IL-1β and pro-IL-18 into their mature active forms [7]. Since sepsis is thought to result from an imbalance between the pro-inflammatory and the anti-inflammatory host responses, there is a growing interest in investigating inflammasome activation in the context of translational studies of bacterial infections [8–10].

Innate immune responses are primarily mediated by myeloid cells, including monocytes. Three monocytes subsets are conventionally distinguished: i) classical monocytes (CM) which are highly phagocytic, ii) intermediate monocytes (ITM) which are characterized by their antigen-presenting capacity properties and their role in T-cell regulation and iii) non-classical monocytes (NCM) which are patrolling cells able to monitor the vascular endothelium to maintain its integrity that can also acquire pro-inflammatory functions [11]. The analysis of monocyte subsets during sepsis began in the 1990s but remained limited thereafter [12]. Interest in this field has since reemerged, driven largely by the potential of monocyte subset profiling to improve diagnostic and prognostic stratification in patients with sepsis. Several studies have reported the increased frequencies of NCM and/or ITM during sepsis [13–15]. In parallel, immature granulocytes and neutrophils subtypes have been the subject of more in‑depth work [16,17]. We recently described a reduced activation potential of the NLRP3 inflammasome in immature neutrophils of patients with severe sepsis-like COVID-19 [18]. Importantly, levels of activated caspase-1 and nigericin-triggered NLRP3 inflammasome activation potential differed markedly among the different circulating myeloid cells [18]. Indeed, NLRP3 activation potential was increased in NCM and was associated with a decrease in NCM frequency and subsequent changes in patient’s oxygen requirement during the days following analysis. These findings provided a proof of concept that the innate immune profiling may help to identify patient at risk of developing severe forms of infections.

However, in ICU translational immunology studies, microbiological data are often inconstantly reported and the study of innate or adaptive immune response according to the etiologic agent involved remains scarce [19]. This limitation also applies the first studies of NLRP3 inflammasome activation through ASC-speck positive monocytes during severe bacterial infections [8,9]. Indeed, several aspects of the immune response usually studied during sepsis are pathogen-dependent such as frequency of myeloid-derived suppressor cells (MDSCs) [20–22]. Efforts made to develop a precision medicine for sepsis patients should therefore incorporate microbiological data because (i) pathogen-specific immune response may require tailored therapeutic strategies (ii) such specificities immune signatures may serve as indirect biomarkers for microbiological diagnosis. Accordingly, we present herein the analysis of circulating myeloid cells subset and their caspase-1 associated activation in the whole blood from patients with either *S. aureus* or *E. coli* bacteremia.

## Methods

### Study design and inclusions

This prospective study was conducted at the University Hospital of Nice, France (Infectious Diseases Departmentand Intensive Care Unit, ICU), as well as at the hospitals of Cannes, France (ICU) and Antibes, France (ICU), enrolling patients, from June 2019 to March 2022. Eligible patients were adults (aged 18 years or older), who presented with a monomicrobial bacteremia caused by either *S. aureus* or *E. coli*, with at least one positive blood culture within 72 hours before inclusion, and a negative SARS-CoV-2 nasopharyngeal PCR test. Exclusion criteria included unremoved urinary tract obstruction, undrained collections within the first 24 hours of management, infections occurring in patients already hospitalized for other reasons, co-infections, pregnancy, breastfeeding, and immunosuppression defined by the use of immunosuppressive treatment, solid organ or hematopoietic stem cell transplantation, chemotherapy-induced neutropenia, or primary immunodeficiency. Healthy control samples were obtained from volunteer blood donors (*n* = 23) at the French Blood Establishment (Établissement Français du Sang, EFS). In accordance with strict ethical guidelines and data privacy regulations, the EFS provides all samples fully anonymized. Consequently, individual demographic and clinical characteristics for these donors were not available.

The study was conducted in accordance with the principles stated in the Declaration of Helsinki [23]. Patients received both oral and written information about the study, and written informed consent was obtained prior to enrollment. The study obtained approval from French ethics committee (Comité de Protection des Personnes Est-II, number 18/11/06/47949) and is registered on clinicaltrial.gov (NCT03869593).

A total of 35 patients were included (Table 1). We thus established a group of 22 patients with *E. coli* bacteremia and a group of 13 patients with *S. aureus* bacteremia. A blood sample was collected at inclusion for flow cytometry analyses. No further samples were taken during subsequent patient management. No therapeutic intervention was associated with the protocol presented in our study. Within both patient groups, we identified patients with sepsis at the time of inclusion based on the Sepsis-3 definition [24]. Time of symptom onset refers to the delay between the first clinical signs reported by the patient related to the portal of entry of the bacteremia, and/or the secondary localization of the bacteremia, and/or the onset of fever.

**Table 1.** Characteristics of the bacteremic patients.

	Total *n* = 35	*E. coli* *n* = 22	*S. aureus* *n* = 13
Age (years)	65.6 (14.9)	71.4 (13.4)	55.9 (12.3)
Male (%)	19 (54%)	10 (45%)	9 (69%)
SAPS II score	27.4 (12.3)	29.4 (12.1)	24.3 (12.5)
SOFA score	2.5 (2.8)	2.8 (3.3)	2 (1.9)
ICU management	4 (11%)	3 (14%)	1 (8%)
Sepsis	19 (54%)	14 (64%)	5 (38%)
Septic shock	2 (6%)	2 (9%)	0 (0%)
Length of hospital stay (days)	17 (17,2)	13.9 (19,9)	22.3 (9,8)
In-hospital mortality	3 (9%)	2 (9%)	1 (8%)
Comorbidities			
Diabete mellitus	13 (37%)	6 (27%)	7 (54%)
Chronic respiratory failure	1 (3%)	0 (0%)	1 (8%)
Chronic heart failure	5 (14%)	2 (9%)	3 (23%)
Hypertension	22 (63%)	16 (73%)	6 (46%)
Chronic renal failure	3 (9%)	1 (5%)	2 (15%)
Chronic liver failure	4 (11%)	2 (9%)	2 (15%)
HIV	1 (3%)	1 (5%)	0 (0%)
Malignancy	7 (20%)	5 (23%)	2 (15%)
Time from symptoms onset to hospitalization (days)	5.9 (9.1)	3.9 (7.4)	8.8 (11)
Blood culture time to positivity (hours)	15.7 (8)	12.3 (3.4)	21.1 (10.2)
Healthcare-associated infection	5(14%)	3(14%)	2(15%)
Primary infection focus			
Urinary tract infection	11 (31%)	10 (45%)	1 (8%)
Biliary	7 (20%)	7 (32%)	0 (0%)
Catheter	4 (11%)	2 (9%)	2 (15%)
Endocarditis	4 (11%)	0 (0%)	4 (31%)
Skin	3 (9%)	0 (0%)	3 (23%)
Unknown	2 (6%)	1 (5%)	1 (8%)
Ascitic fluid infection	2 (6%)	2 (9%)	0 (0%)
Bone and joint infection	2 (6%)	0 (0%)	2 (15%)
Blood count			
WBC (G/L)	11.3 (5.6)	9.7 (4.1)	14 (6.8)
Neutrophils (% of WBC)	77.1 (13.4)	78.5 (13.9)	74.8 (12.7)
Eosinophils (% of WBC)	1.3 (1.9)	1 (1)	1.8 (2.7)
Basophils (% of WBC)	0.3 (0.2)	0.3 (0.3)	0.3 (0.2)
Lymphocytes (% of WBC)	12.3 (9.8)	11.9 (10.4)	12.9 (9)
Monocytes (% of WBC)	7.8 (3.7)	7.3 (4)	8.4 (3.1)
C-reactive protein at inclusion (mg/l)	137.1 (92.8)	130.4 (87.6)	147.8 (103.6)

Continuous variables are expressed as mean (standard deviation), categorical variables are described as frequencies (percentage).

### Flow cytometry preparation and staining

Blood samples were collected from patients via peripheral venous puncture into citrate tubes and analyzed within 24 hours. Whole blood was diluted in RPMI medium 1/1 and subsequently incubated for 30 minutes with the FAM-FLICA caspase-1 kit (Immuno-Chemistry Technologies), containing the irreversible intracellular marker FAM-YVAD-FMK, which fluoresces upon excitation at 488 nm. Viobility™ (Miltenyi Biotec) staining, an intracellular marker, was used to assess cell death. MFI value for FAM-FLICA in unstimulated condition represents basal caspase-1 activation.

Cells were further incubated for 10 minutes at room temperature, shielded from light, with recombinant surface membrane antibodies (Miltenyi Biotec) at a 1/100 dilution, including CD45-VioGreen (clone REA747), CD14 APC-Vio770 (clone REA599), CD16-phycoerythrin (PE) (clone REA423), CD66b PE-Vio770 (clone REA306), HLA-DR PE-Vio615 (clone REA805), CD10 APC-Vio770 (clone REA877), and Siglec-8-PE-Vio615 (clone REA1045). One non-recombinant antibody, CD15 APC (Miltenyi Biotec, clone VIMC6), was also used, with prior incubation with an Fc receptor blocking reagent (Miltenyi Biotec) for 10 minutes when using these antibodies.

Red blood cell lysis was performed using osmotic lysis buffer (BD Biosciences, BD Pharm Lyse Buffer) following the supplier’s instructions. Cells were fixed in 4% paraformaldehyde for 10 minutes.

### Flow cytometry analysis

The marked cells were then analyzed using a MACSQuant 10 flow cytometer (Miltenyi Biotec). A minimum of 80,000 leukocytes (defined by CD45^+^ cells) per condition were recorded. Raw cytometric data were analyzed with FlowJo v10.8.1. The gating strategy is presented in Supplementary Figure S1. After eliminating cellular debris and doublets, leukocytes were identified as CD45^+^ cells. Granulocytes were selected based on CD66b expression, and monocytes were identified as CD66b^−^ and CD14^+^ and further divided into three subpopulations: classical monocytes (CD14^high^ CD16^−^), intermediate monocytes (CD14^high^ CD16^+^), and non-classical monocytes (CD14^low^ CD16^+^). Granulocytes were also divided into three subpopulations, including mature neutrophils (CD66b^+^ CD16^high^), eosinophils (CD66b^+^ CD16^low^ Siglec-8^+^), and immature neutrophils (CD66b^+^ CD16^low^ CD15^+^ CD10^−^). For all monocytic or granulocytic subpopulations, the median fluorescence intensity (MFI) of FAM-FLICA (in FITC) was measured.

### Statistical analyses

Statistical analysis of clinical and flow cytometry data was performed using GraphPad Prism v 9.4. Depending on the data distribution, parametric tests such as Student’s t-test, one-way ANOVA, two-way ANOVA, or non-parametric tests like Mann-Whitney and Kruskal-Wallis were employed. In cases where Kruskal-Wallis tests were significant (*p* < 0.05), multiple comparisons were assessed using the uncorrected Dunn’s posttest, which was justified for planned post-testing exclusively within the *E. coli* and *S. aureus* groups. Details of the tests are available in the figure legends.

## Results

### NCM count and associated caspase-1 activation are increased during *E. coli* bacteremia but not *S. aureus* bacteremia

During bacteremia, circulating leukocytes are exposed to various pathogenic bacteria that reached the blood. Considering that Gram-positive and Gram-negative bacteria harbor different PAMPs and behave differently during infection, we hypothesize that the patient’s myeloid cells flow cytometry profiling and caspase-1 signature would differ according to the infecting pathogen and could provide a pathogen-specific signature for each type of infection. To address this question experimentally we prospectively recruited adult patients with a monomicrobial infection caused by either *S. aureus* or *E. coli*, the two most common bacterial pathogens isolated during bacteremia in clinics, whose clinical characteristics are summarized in Table 1.

Peripheral blood cells of patients with *E. coli* or *S. aureus* bacteremia were prepared for flow cytometry as previously described [18] and stained for CD45, CD14, CD16, CD66b, CD15, CD10, Siglec-8 markers together with the FAM-FLICA probe to detect enzymatically active caspase-1. Healthy donors (*n* = 23) were included and analyzed in parallel using identical acquisition and analysis settings (Supplementary Figure S1). This approach enables the identification and characterization of the 3 classes of monocytes (CM, ITM, and NCM), both quantitatively, by measuring their relative frequencies (Figure 1(A-C)) and functionally, by measuring their relative caspase-1 activation through FAM-FLICA median fluorescence intensity (MFI) (Figure 1(G-I)).

**Figure 1.**
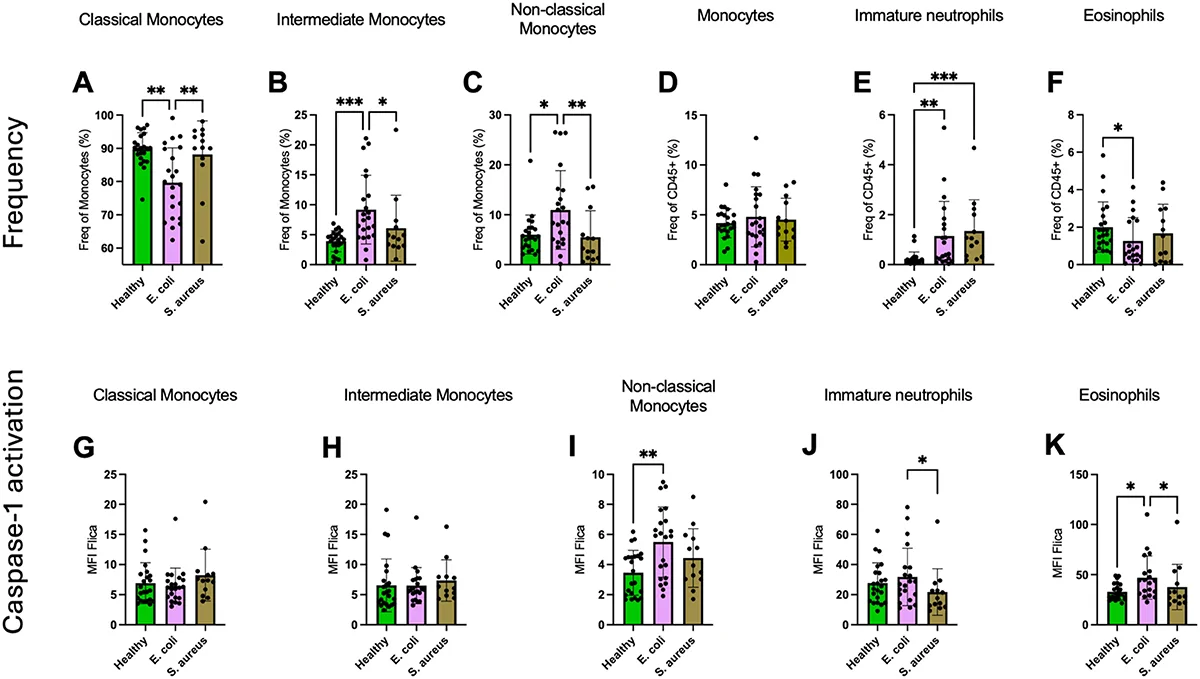
Circulating myeloid cells and associated caspase-1 activation according to the bacteria isolated in blood cultures. Whole peripheral blood cells of healthy donors (*n* = 23) or patients with *E. coli* or *S. aureus* bacteremia were stained for CD45, CD14, CD16, CD66b, CD15, CD10, Siglec-8 markers and for active caspase-1 (detected using the FAM-FLICA probe). Cells were immunophenotyped by flow cytometry. Leukocytes were defined as CD45^+^. The frequency of monocytes subsets among whole monocytes was analyzed: CD14^high^CD16^−^ for classical monocytes (A), CD14^high^CD16^+^ for intermediate monocytes (B), and CD14^dim^CD16^+^ for non-classical monocytes (C). The frequency of monocytes (CD14^+^) among leukocytes is also displayed (D). CD66b^+^CD16^dim^ cells were analyzed for CD10 (marker of mature neutrophil) and CD15 (neutrophil marker) and the frequency of CD66b^+^CD16^dim^CD15^+^CD10^−^ immature neutrophils among leukocytes was determined (E). CD66b^+^CD16^dim^ cells were analyzed for CD66b and Siglec-8 (eosinophil marker) and the frequency of CD66b^+^CD16^dim^Siglec8^+^ eosinophils among leukocytes was determined (F). Except for classical monocytes, cells were analyzed for the median fluorescence intensity (MFI) of FAM-FLICA corresponding to the activation of caspase-1 (G-K). One outlier with a very high FLICA MFI value was excluded from the intermediate monocyte subgroup (*S. aureus*, non-sepsis). Graphs show mean +/-SD. Data were analyzed with Kruskal-Wallis nonparametric test. If it showed a statistically significant (*p* < 0.05) difference, multiple comparisons were tested by the uncorrected Dunn’s test. Ns *p* > 0.05; * *p* ≤ 0.05; **p ≤ 0.01; ***p ≤ 0.001; ****p ≤ 0.0001.

Interestingly, we measured a specific increase of ITM and NCM during *E. coli* bacteremia whereas no such increase was observed in the blood of patients with *S. aureus* bacteremia (Figure 1(A-C)). The frequency of ITM among monocytes was 3.9 % in healthy donors, 9.2% for *E. coli* (*p* = 0.0002) and 6.1 % for *S. aureus* (*p* = 0.32) and frequency of NCM was 6.0%, 10.9% (*p* = 0.04) and 5.5% (*p* = 0.39), respectively (Figure 1(B,C)). Consistent with an increased frequency of NCM and ITM, we observed a proportionally decreased frequency of CM in *E. coli* bacteremic patients (Figure 1(A)). The increase in frequency was specific to ITM and NCM since the rate of CM was decreased in *E coli* or not affected in *S aureus* infected patients compared to healthy donors. The whole monocytes count (% CD45+ cells, Figure 1(D)) did not show any statistically differences across the groups highlighting the importance of monocytes subset analysis rather than total monocyte counts. Importantly, the functional analysis of caspase-1 showed a median fluorescence intensity (MFI) of activated caspase-1 detection in NCM significantly elevated specifically during *E. coli* bacteremia compared to healthy donor whereas no increase was observed in patients with *S. aureus* bacteremia (Figure 1(I)).

Our flow cytometry analysis of granulocytes revealed an increased frequency of immature neutrophils during both *E. coli* and *S. aureus* bacteremia (Figure 1(E)). We measured a slightly but statistically significant decreased MFI of activated caspase-1 in immature neutrophils for *S. aureus* compared to *E. coli* (Figure 1(J)). In addition, patients with *E. coli* bacteremia displayed reduced eosinophils counts associated with an increased caspase-1 activation in eosinophils (Figure 1(F, K)).

We observed that patients with *E. coli* bacteremia were older than patients with *S. aureus* bacteremia (mean age of 71 *vs* 56 years) and presented a shorter time since the onset of symptoms at enrollment (mean time of 3.9 *vs* 8.8 days). We did not identify any statistically significant correlation between age and the myeloid populations nor between the time since the onset of symptoms and myeloid populations or the related activation of caspase-1 that could explain these differences (Supplementary Figures S2 and S3). Although a weak inverse correlation was identified between age and a FLICA MFI of ITM, suggesting that age may have attenuated an increase in caspase-1 activation during *E. coli* bacteremia (Figure 1(H)), the increase in caspase-1 activation observed in NCM during *E. coli* bacteremia was independent of patient age (Supplementary Figure S2).

### Disease severity influences frequency of circulating myeloid cells and associated caspase-1 activation during bacteremia

We next investigated whether these observed differences between the recorded responses against *E. coli* and *S. aureus* were associated to the infecting bacteria or were influenced by other factors. Importantly, the profile of patients with *S. aureus* and *E. coli* bacteremia differs on one key point, which is disease severity: more patients with *E. coli* bacteremia had sepsis (Table 1). Because the dysregulated host’s response during sepsis may be associated with variations in circulating myeloid cells and associated caspase-1 activation, we stratified patients according to the presence or absence of sepsis, defined by SOFA score ≥ 2 according to the SEPSIS-3 criteria [24] regardless of the bacteria involved.

While the frequency of CM remains stable (Figure 2(A)), we measured an increase in ITM frequency specifically in patients presenting sepsis but not in bacteremic patients without sepsis criteria (Figure 2(B)). In contrast, we found no difference associated with the NCM frequency (Figure 2(C)). Conversely, despite the absence of change in NCM frequency, the MFI of activated caspase-1 in NCM was significantly elevated in both types of patients compared to healthy donors (Figure 2(H)). The magnitude of increase was similar between sepsis and non-sepsis patients. The Caspase-1 activation was not modified in CM or ITM of bacteremic patients in the two types of host’s response (Figure 2(F, G)). The frequency of Immature neutrophils was non‑significantly increased in sepsis patients compared to non-sepsis patients (mean 1.31% vs 1.12%, *p* = 0.11) (Figure 2(D)). Likewise, caspase-1 activation in immature neutrophils did not differ between sepsis and non-sepsis patients (Figure 2(I)). However, eosinophils count of sepsis patients was drastically decreased compared to healthy donors: 0.94% vs 2.0% (Figure 2(E)). Conversely, eosinophils frequency in bacteremic patients without sepsis was not reduced compared to healthy donor (Figure 2(E)). Interestingly, the decrease of eosinophils in sepsis patients was associated with an increase in caspase-1 activation (Figure 2(J)).

**Figure 2.**
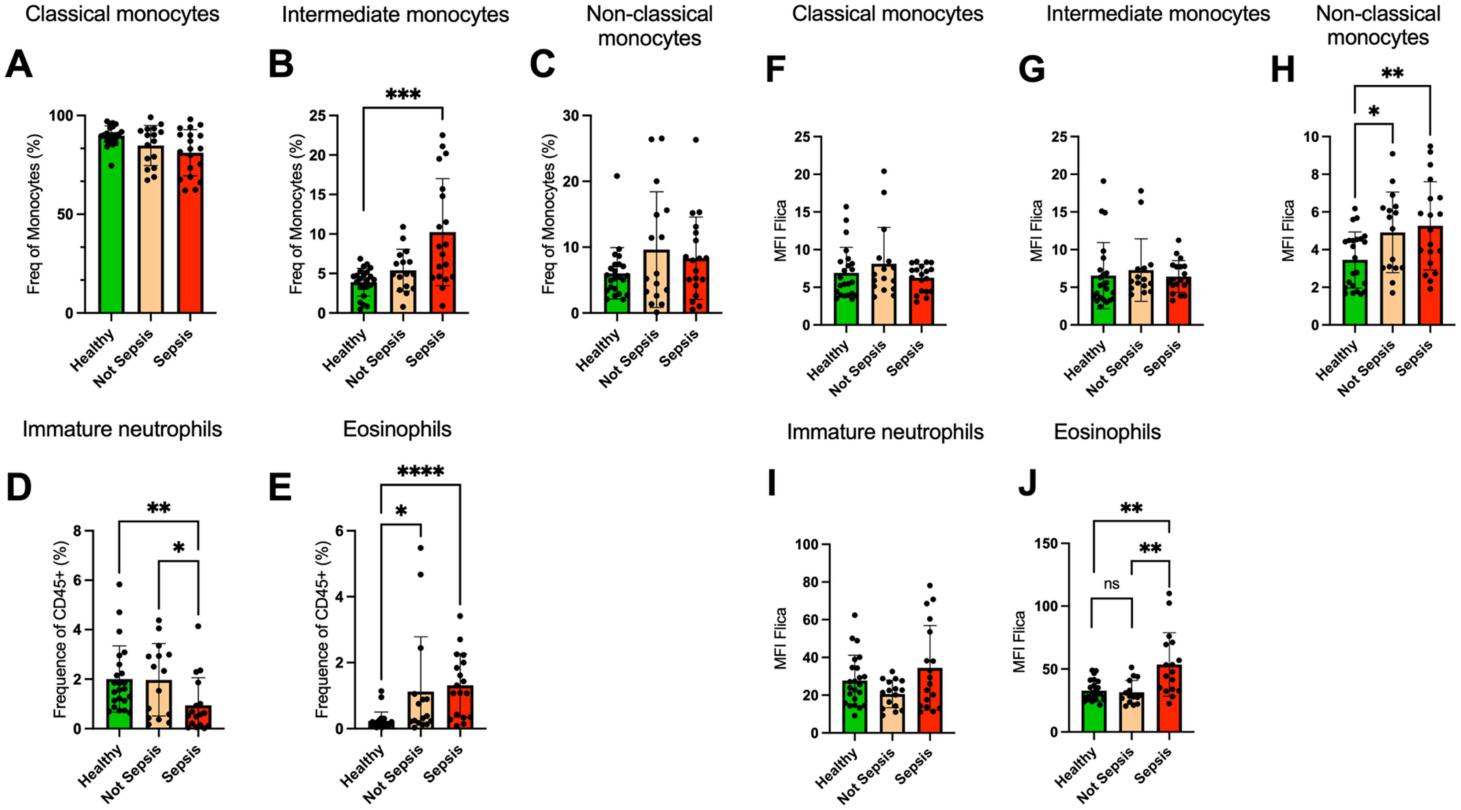
Circulating myeloid cells and caspase-1 activation according to disease severity during bacteremia. Whole peripheral blood cells of healthy donors (*n* = 23) or patients with sepsis or no sepsis during the course *Escherichia coli* and *Staphylococcus aureus* bacteremia were stained for CD45, CD14, CD16, CD66b, CD15, CD10, Siglec-8 markers and for active caspase-1 (detected using the FAM-FLICA probe). Sepsis was defined by the sepsis-3 criteria, which are based on sepsis-related organ failure assessment (SOFA) score. The cells were immunophenotyped by flow cytometry. Leukocytes were defined as CD45^+^. The frequency of monocytes subsets among whole monocytes was analyzed: CD14^high^CD16^−^ for classical monocytes (A), CD14^high^CD16^+^ for intermediate monocytes (B) and CD14^dim^CD16^+^ for non-classical monocytes (C). CD66b^+^CD16^dim^ cells were analyzed for CD10 (marker of mature neutrophil) and CD15 (neutrophil marker) and the frequency of CD66b^+^CD16^dim^CD15^+^CD10^−^ immature neutrophils among leukocytes was determined (D). CD66b^+^CD16^dim^ cells were analyzed for CD66b and Siglec-8 (eosinophil marker) and the frequency of CD66b^+^CD16^dim^Siglec8^+^ eosinophils among leukocytes was determined €. Those five types of cells were analyzed for the median fluorescence intensity (MFI) of FAM-FLICA corresponding to the activation of caspase-1 (F-J). One outlier with a very high FLICA MFI value was excluded from the intermediate monocyte subgroup (*S. aureus*, non-sepsis). Graphs show mean +/-SD. Data were analyzed with Kruskal-Wallis nonparametric test. If it showed a statistically significant (*p* < 0.05) difference, multiple comparisons were tested by the uncorrected Dunn’s test. Ns *p* > 0.05; * *p* ≤ 0.05; **p ≤ 0.01; ***p ≤ 0.001; ****p ≤ 0.0001.

### NCM‑associated caspase 1 modifications during *E. coli* bacteremia are driven by the involved bacteria not disease severity

We have shown herein that both the pathogen and sepsis severity are associated with changes in circulating myeloid cell subpopulations and caspase-1 activation. To determine which of these factors predominantly influence on the changes in each circulating myeloid cell population, we stratified the patients according to both microbiological data and the disease severity (Figure 3(A-I)). We found that difference of ITM frequency between *E. coli* and *S. aureus* bacteremic patients were mainly driven by the intensity of pathogen-related host response. Patients with *E. coli* sepsis exhibited significantly higher ITM frequency than those with *S. aureus* sepsis (*p* = 0.011) whereas no difference was observed in non-sepsis patients (Figure 3(B)). Within the *E. coli* group, ITM frequency in non-sepsis patients (5.4%) was below to those sepsis patients (11.4%), *p* = 0.038 (Figure 3(B)).

**Figure 3.**
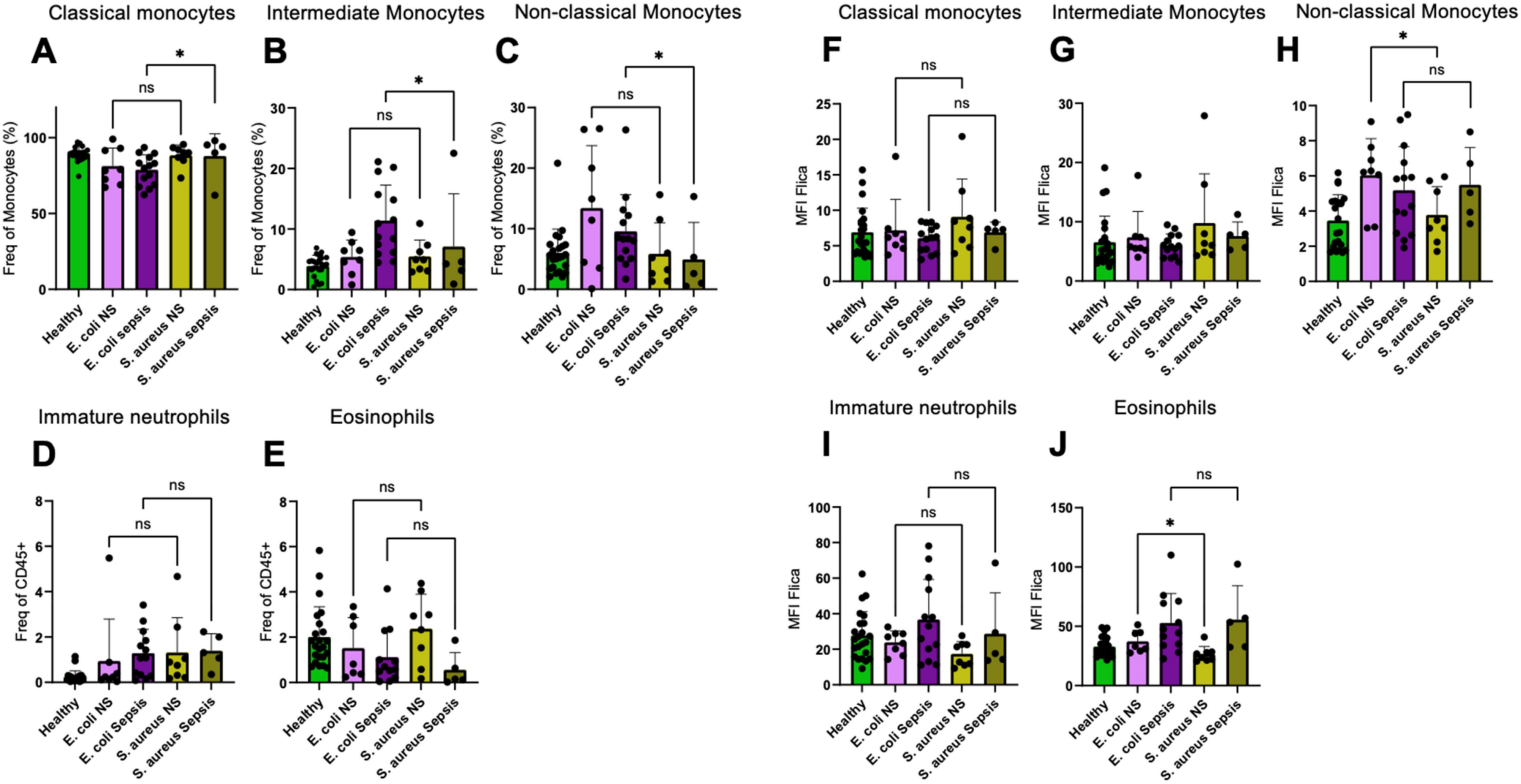
Circulating myeloid cells and caspase-1 activation according to disease severity and etiologic agent during bacteremia. Whole peripheral blood cells of healthy donors (*n* = 23) or patients with sepsis or no sepsis during the course *Escherichia coli* and *Staphylococcus aureus* bacteremia were stained for CD45, CD14, CD16, CD66b, CD15, CD10, Siglec-8 markers and for active caspase-1 (detected using the FAM-FLICA probe). Sepsis was defined by the sepsis-3 criteria, which are based on sepsis-related organ failure assessment (SOFA) score. The cells were immunophenotyped by flow cytometry. Leukocytes were defined as CD45^+^. The frequency of monocytes (represented as percentages) subsets among whole monocytes was analyzed: CD14^high^CD16^−^ for classical monocytes (A), CD14^high^CD16^+^ for intermediate monocytes (B) and CD14^dim^CD16^+^ for non-classical monocytes (C). CD66b^+^CD16^dim^ cells were analyzed for CD10 (marker of mature neutrophil) and CD15 (neutrophil marker) and the frequency of CD66b^+^CD16^dim^CD15^+^CD10^−^ immature neutrophils among leukocytes was determined (D). CD66b^+^CD16^dim^ cells were analyzed for CD66b and Siglec-8 (eosinophil marker) and the frequency of CD66b^+^CD16^dim^Siglec8^+^ eosinophils among leukocytes was determined (E). Those five types of cells were analyzed for the median fluorescence intensity (MFI) of FAM-FLICA corresponding to the activation of caspase-1 (F-J). One outlier with a very high FLICA MFI value was excluded from the intermediate monocyte subgroup (*S. aureus*, non-sepsis). Graphs show mean +/-SD. Data were analyzed with Kruskal-Wallis nonparametric test. If it showed a statistically significant (*p* < 0.05) difference, multiple comparisons were tested by the uncorrected Dunn’s test.

In addition, we observed that NCM frequency was increased in *E. coli* bacteremia over *S. aureus* bacteremia (Figure 3(C)), significantly in the subgroup of sepsis patients (9.5% vs 4.9 %, *p* = 0.041) and with a trend in non-sepsis patients (13.4% vs 5.8%, *p* = 0.094). Furthermore, there was no difference of NCM frequency between non-sepsis and sepsis patients during *E. coli* BSI *p* = 0.87. This data supports the hypothesis that the differences observed among NCM are mainly pathogen-driven rather than driven by disease severity. Similarly, the elevation of caspase-1 activation in NCM during *E. coli* bacteremia is not driven by disease severity (Figure 3(H)). Proportion of CM among monocytes was higher in *S. aureus* sepsis patients compared to *E. coli* sepsis patients (Figure 3(A)) and accordingly to Figures 1(G) and 2(I) we did not detect any significant difference in caspase-1 activation in CM (Figure 3(F)).

Our analysis of immature neutrophils did not allow us to distinguish the relative importance of disease severity and pathogen identity to either their frequency or caspase-1 activation, suggesting that both factors may contribute to this phenotype (Figure 3(D, I)).

Regarding eosinophils, the comparisons provided in Figure 3(E) remain inconclusive, suggesting that disease severity (Figure 2(E)) rather than pathogen identity is the primary driver of the decreased eosinophils count recorded in this study. Likewise, as for immature neutrophils, we were unable to distinguish the respective contributions of the etiologic agent and disease severity to the differences in eosinophils’ caspase-1 activation (Figure 3(J)).

Since NCM frequency appeared to be primarily influenced by the infecting bacteria and could help to distinguish between *E. coli and S. aureus*, we next asked whether this signature could be also help to distinguish bacterial from viral infections. To address this question, we compared NCM frequencies with those measured in patients with severe and critical COVID-19 patients with similar settings and inclusion period (clinicaltrials.gov NCT04385017).

Strikingly, NCM frequency was markedly lower in severe and critical COVID-19 than in *S. aureus* an *E. coli* bacteremia (Figure 4(A, B)). While the caspase 1 activation did not differ significantly between bacterial and viral infection.

**Figure 4.**
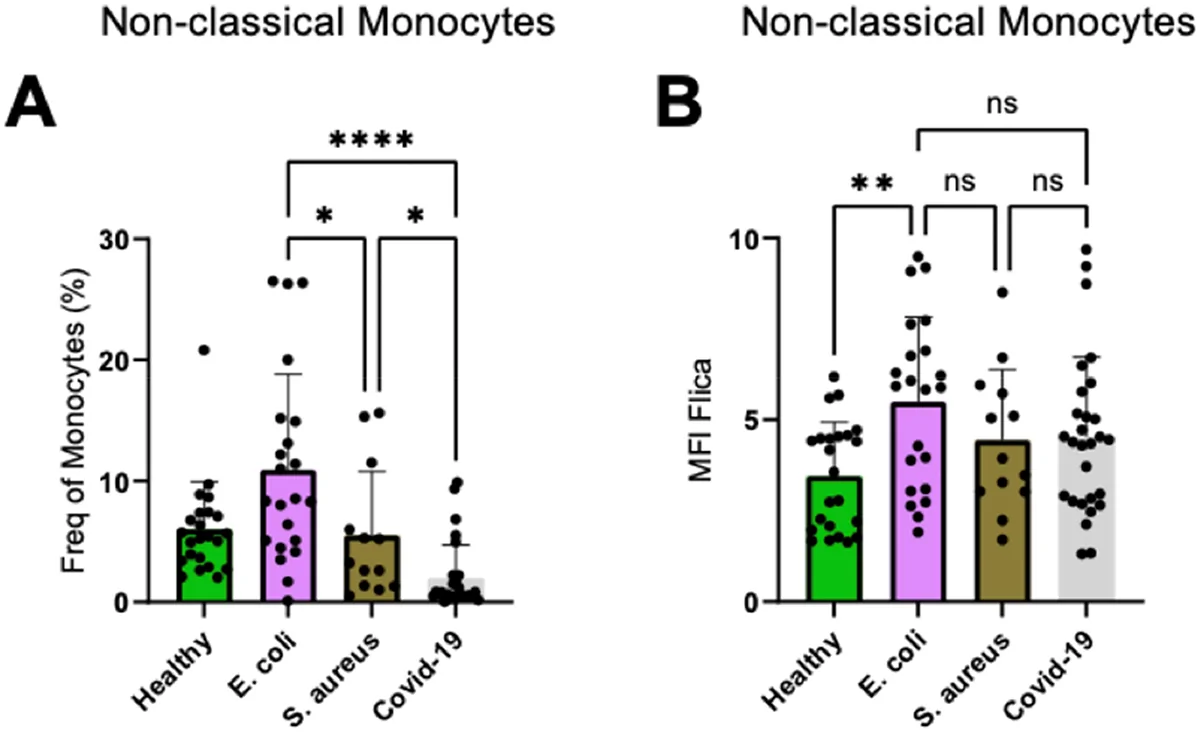
NCM monocytes count and caspase-1 activation between bacteremia and severe or critical SARS-CoV-2 infection. Whole peripheral blood cells of healthy donors (*n* = 23) or patients with *E. coli* or *S. aureus* bacteremia, or patients with severe or critical SARS-CoV-2 infection were stained for CD45, CD14, CD16, CD66b, CD15, CD10, Siglec-8 markers and for active caspase-1 (detected using the FAM-FLICA probe). The COVID-19 patients represented in the graph come from a study conducted by our team with an inclusion period overlapping with this study and with a similar methodology (clinicaltrials.Gov NCT04385017). Cells were immunophenotyped by flow cytometry. Leukocytes were defined as CD45^+^. The frequency of non-classical monocytes CD14dimCD16+ among whole monocytes was analyzed (A). Non-classical monocytes were analyzed for the median fluorescence intensity (MFI) of FAM-FLICA corresponding to the activation of caspase-1 (B). Graphs show mean +/-SD. Data were analyzed with Kruskal-Wallis nonparametric test. If it showed a statistically significant (*p* < 0.05) difference, multiple comparisons were tested by the uncorrected Dunn’s test. Ns *p* > 0.05; *p ≤ 0.05; **p ≤ 0.01; ***p ≤ 0.001; ****p ≤ 0.0001.

## Discussion

Our work provides, to the best of our knowledge, the first insights into pathogen-specific alterations in circulating myeloid cell composition and associated caspase-1 activation in the blood of bacteremic patient’s blood infected with the two most common bacterial pathogen responsible for BSI, namely *E. coli* and *S. aureus*. We showed that the increased ITM correlates with both the bacterial genus and the disease severity. Conversely, we show that the increase in NCM frequency and increased caspase-1 activation appeared to be specific of the pathogen. The decrease in eosinophils count during *E. coli* BSI and SAB correlated with disease severity. Notably, the distinct profiles of *E. coli* vs *S. aureus* with respect to caspase-1 activation in NCM were predominantly identified within the cohort of non-sepsis patients.

When studying the evolution of monocytes subsets following experimental endotoxemia, Thaler *et al*. recorded an increase of ITM frequency 6 hours after the beginning of the experiment [25]. NCM showed a trend toward an increased frequency 24 hours later, consistent with the fate of ITM. It is also crucial to consider that the mean interval between symptom onset and sampling was 6 days in our cohort. Such a delay may have allowed the transition from ITM to NCM [26,27].

Consistent with our data, a previous study reported increases in both NCM and ITM during BSI [13]. However, this study did not stratify patients according to bacterial species or infection source. Monocytes subset dynamics has been studied specifically during Gram-negative sepsis including patients with pyelonephritis, acute intra-abdominal infections and primary bacteremia [14]. CM and ITM were pooled together in their analysis and were elevated compared to healthy controls. NCM were likewise increased from Day 1 and interestingly the absolute number of NCM was significantly higher in survivors than in non-survivors 3 days after enrollment. Most of our patients were managed outside the ICU with a relatively low mean SOFA score (2.5). By contrast, the study by Hortová-Kohoutková *et al*., which included patients with septic shock with a relatively high mean SOFA score (11.5) caused by infection from various sources and etiologic agents, did not identify a difference in NCM numbers between survivors and patients who died early [15]. In *E. coli* BSI we found a trend toward higher frequency of NCM in non-sepsis patients compared to sepsis patients (Figure 3(C)). Further studies with larger cohort of patients will be needed to determine whether the magnitude of NCM elevation reflects, or contribute to, an appropriate host response to *Enterobacteriaceae* bacteremia.

The hypothesis that pathogen-related modifications in circulating myeloid cells precede organ failure is not supported by our analysis of duration of symptoms, which did identify a significant influence of this variable. Moreover, NCM have the longest lifespan among monocytes subsets (approximately 7 days) [26] suggesting that despite a longer symptoms duration observed in patients with *S. aureus* bacteremia, the timing of sampling was unlikely to have masked the NCM elevation. One possible explanation for the NCM elevation specifically during *E. coli* bacteremia is that tumor necrosis factor a (TNFa) has been shown *in vitro* to promote monocytes differentiation toward NCM phenotype [28,29]. Furthermore, TNFa level isusually detected at higher levels during Gram negative compared to Gram positive bacteremia [30].

Inflammasome activation has also been previously investigated during sepsis in patients with an Inflammasome activation (using ASC-speck positive leukocyte detection) shown mainly in CM 6 days after enrollment [8]. Despite including patients with different infection sources, they found that low ASC-speck positive monocytes were associated with 90-day mortality. The authors proposed that this offbeat detection of inflammasome activation can be considered a good indicator of the maintenance or resolution in an sepsis-induced immunoparalysis state [31]. Conversely Martinez-Garcia *et al*. identified increased numbers of ASC-speck positive monocytes on the first day after enrollment in patient with sepsis [9]. These two studies are somehow consistent since elevated ASC-speck positive monocytes were associated to a favorable outcome in both cases. However, the difference in sampling time points, together with methodological differences (whole blood analysis (Cui *et al*.) vs analysis of PBMC after Ficoll gradient (Martinez-Garcia *et al*.)), warrants caution when comparing these studies. Using the FLICA probe, we identified caspase-1 activation in NCM from bacteremic patients, most of whom were managed outside the ICU and had a low mortality rate, as early as the first day following positive blood culture positivity. Moreover, the greatest caspase-1 activation measured in NCM was correlated with the non-sepsis status which is consistent with the two studies discussed above and made and support the possibility that activated caspase-1 in NCM may represent a biomarker of an appropriate host response to *Enterobacterales* bacteremia.

A limitation of our study is that inflammasome activation was assessed by intracellular detection of enzymatically active caspase-1 using the FAM-FLICA probe. Although this approach is widely accepted for evaluating inflammasome activation at the single-cell level, complementary biochemical analyses of sorted myeloid cell subsets, such as immunoblotting for caspase-1 processing or gasdermin D cleavage, would provide additional validation. Future studies using alternative experimental models and larger numbers of purified cells will be required to further validate these findings using complementary biochemical approaches.

Eosinopenia has already been described during bacteremia and has even been associated to unfavorable outcome [32]. In a human model of endotoxemia, within hours, circulating mature eosinophils and blood eosinophils rapidly declined within hours whereas eosinophils precursor remain unaffected [33]. Based on a reduced CD49D expression, the authors suggested the possibility of a tissue-homing mechanism. However, they did not identify any activation or degranulation of the blood eosinophils based on surface marker analysis. In contrast, we found an increased caspase-1 level in the remaining circulating eosinophils suggesting that these cells undergo inflammasome activation during bacteremia. Further studies will be required to determine the role of the functional significance of this response, nevertheless our observation suggests that this feature might be used as a biomarker to distinguish between different types of pathogens.

Here we have shown that NCM and eosinophils display opposite changes in frequency during bacteremia, whereas both cell populations exhibit increased caspase-1 activation.

Circulating myeloid cells were analyzed directly on whole blood, rather than after Ficoll purification, a procedure known to artificially decrease in CM frequency while increasing NCM frequency [34].

The originality of our cohort lies in the inclusion of patients predominantly managed outside the ICU and with a low mortality rate among the 35 patients. This recruitment likely enabled favorable conditions for identifying pathogen-specific immune signatures without confounding effects of a cytokine storm associated with a septic shock and severe dysregulated immune responses. Future studies will have to include patients with a broader range of clinical outcomes during bacteremia to determine whether circulating myeloid cells level and the associated caspase-1 activation relationship can predict disease progression. These studies might compare *E. coli* and *S. aureus* patients with similar primary infection sites, although such comparisons may be challenging because of the distinct pathophysiology and epidemiology of these pathogens in community-acquired infections.

An additional limitation of our study is that pathogen classification relied on routine blood culture results. Although patients were categorized according to the pathogen identified by conventional microbiological diagnosis, a culture-confirmed bloodstream infection does not necessarily represent a truly monomicrobial infection. Recent metataxonomic and metagenomic studies have suggested that BSI may involve more complex microbial communities than those detected by routine culture-based methods [35]. Consequently, the immune signatures identified in our study may reflect the host response to the dominant cultured pathogen with a potential contribution of additional microorganisms that remained undetected by standard diagnostics. Nevertheless, because blood culture remains the current clinical gold standard for bloodstream infection diagnosis and therapeutic decision-making, our findings remain directly relevant to routine clinical practice. Future studies combining high-resolution microbiological approaches with immune phenotyping will be important to determine how polymicrobial signatures influence circulating myeloid cell composition and inflammasome activation.

Here, we provide new insights into prerequisites to improve inflammasome activation evaluation in patients: homogeneous bacterial etiologies, in-depth analysis of monocytes subtypes and subgroups of different disease severity. We show that combining an immunophenotyping with a biomarker of the innate immune activation enables discrimination between *E. coli* and *S. aureus* using peripheral patient’s blood samples. These findings provide a framework for future studies investigating pathogen-specific immune response and may ultimately facilitate the development of more precise immunomonitoring strategies in the era of immunomodulatory therapies.

## Supplementary Material

Supplementary 20260713.docx

## Data Availability

The data that support the findings of this study are openly available in « Dataset of the paper : Differences in circulating levels of Non-Classical Monocytes and associated caspase 1 activation during Staphylococcus aureus and Escherichia coli bacteremia » at https://doi.org/10.5281/zenodo.18803777 [36].

## Open scholarship

This article has earned the Center for Open Science badge for Open Materials. The materials are openly accessible at https://zenodo.org/records/18803777
