# Supplementary material for: Differences in circulating levels of non-classical monocytes and associated caspase 1 activation during Staphylococcus aureus and Escherichia coli bacteremia
Source: Virulence. 2026 Nov 1;17(1):2730393. doi: 10.1080/21505594.2026.2730393 (PMC13733151; doi:10.1080/21505594.2026.2730393)
Supplement: Supplementary file 1 [file KVIR_A_2730393_SM2610.docx]

**Supplementary Figure 1 Gating Strategy.** Gating strategy of one healthy donor **(A)** and one *E. coli* bacteremic patient **(B)**. NCM, Non-classical monocytes. CM, Classical monocytes. ITM, Intermediate monocytes.

**A**

**B**

**Supplementary Figure 2**. Correlation between age and myeloid populations frequencies and Caspase-1 activation. Simple linear correlation was performed with age in X-axis and other values in Y-axis. Goodness of fit was represented with R² on the graph only when it was a significant correlation.

**Supplementary Figure 3.** Correlation between the delay from symptom onset and monocyte populations frequencies and caspase-1 activation. Simple linear correlation was performed the delay (in days) from symptoms onset and admission in X-axis and other values in Y-axis. Goodness of fit was represented with R² on the graph only when it was a significant correlation. For all populations tested here, no significant association was found between delay from symptoms onset and frequency or MFI values. Nine patients were excluded from this analysis because the delay was not available in the medical file for 7 patients and 2 were excluded because they were outliers with 30 and 40 days of delay.
